# Protective effect of madecassoside on H_2_O_2_-induced oxidative stress and autophagy activation in human melanocytes

**DOI:** 10.18632/oncotarget.17654

**Published:** 2017-05-07

**Authors:** Yuting Ling, Qingli Gong, Xixi Xiong, Li Sun, Wene Zhao, Wenyuan Zhu, Yan Lu

**Affiliations:** ^1^ Department of Dermatology, The First Affiliated Hospital of Nanjing Medical University, Nanjing, Jiangsu Province, 210029, P.R. China; ^2^ Department of Analysis and Testing Center, Nanjing Medical University, Nanjing, Jiangsu Province, 210029, P.R. China

**Keywords:** madecassoside, oxidative stress, autophagy, mitochondria, vitiligo

## Abstract

**Background:**

*Centella asiatica* (L.) Urb. is a traditional Chinese medicine that has many medical applications, including wound healing and anti-oxidation. Some traditional Chinese Medicine doctors have found that it has therapeutic effects for external use in the repigmentation of vitiligo and post-inflammatory hypopigmentation. This study was designed to evaluate the effects of madecassoside, a major bioactive component of *C. asiatica*, on oxidative stress in human melanocytes and its possible mechanism of action.

**Results:**

In H2O2-induced oxidative conditions, madecassoside inhibited melanocyte dendrite retraction, improved MMP and reduced the accumulation of [Ca2+]i in a concentration-dependent manner. Observations by TEM showed that madecassoside attenuated the damage of mitochondria in human melanocytes caused by oxidative stress. Furthermore, autophagy activation was demonstrated by AO staining and an increased LC3-II/LC3-I ratio.

**Materials and Methods:**

Normal human melanocytes were treated with 0.01 mM H2O2 and varying concentrations of madecassoside (0, 10, 50, 100 μg/mL). Subsequently, the retraction velocity of melanocyte dendrites was assessed. Determination of mitochondrial membrane potential (MMP, ΔΨm) was performed by flow cytometry and intracellular calcium ([Ca^2+^]i) level were measured. Alterations of mitochondrial ultrastructure were observed by transmission electron microscopy (TEM). Acridine orange (AO) staining was used to measure autophagy. The LC3-II/LC3-I ratio, an indicator of autophagosome formation, was analyzed by western blot.

**Conclusions:**

These results demonstrate the antioxidative effect of madecassoside on human melanocytes subjected to oxidative damage via the activation of autophagy. Moreover, madecassoside could be a promising treatment for vitiligo mainly caused by oxidative stress.

## INTRODUCTION

Vitiligo is an acquired pigmentary disorder of the skin and mucous membranes, and is characterized by a chronic and progressive loss of melanocytes from the epidermis and follicular reservoir. Several hypotheses have been proposed to explain the pathomechanism of vitiligo, including the neural theory, an impaired redux status and autoimmunity [1]. Recently, oxidative stress has been suggested to be the initial pathogenic event in melanocyte degeneration and evidence has been presented for very high levels of H_2_O_2_ (1 mM) and peroxynitrite in the epidermis of patients with active vitiligo disease, concomitant with reduced levels and activity of catalase [2–4].

Melanocytes dendrites have crucial roles in the adhesion, migration, and melanosome transfer. In human skin, each melanocyte makes contact with several keratinocytes via their dendrites. A new theory called ‘melanocytorrhagy’ has been proposed that depigmentation in vitiligo results from a chronic detachment and subsequent transepidermal elimination of melanocytes, which is possibly related to increased susceptibility to mechanical and other types of stresses like oxidative stress [5]. Schallreuter et al. [4]. Proved that the addition of H_2_O_2_ to established cultures of normal human melanocytes induced a loss of dendricity and in some cases caused melanocyte detachment. Attachment during dendrite outgrowth requires extracellular Ca^2+^, and H_2_O_2_ induces an impaired uptake and efflux of Ca^2+^ in melanocytes that hinders the dendrite outgrowth and elongation [6].

Mitochondria are the main targets of reactive oxygen species (ROS) which can result in changes of mitochondrial structure and function, including Ca^2+^ homeostasis, cellular redox state regulation and so on [7]. Picardo’s group has demonstrated an imbalance of antioxidants in the peripheral blood mononuclear cells of active vitiligo patients, which appeared to be due to a mitochondrial impairment[8]. In this regard, it is likely that mitochondria play a pivotal role in the etiopathogenesis of vitiligo.

*Centella asiatica* (L.) Urb. is a perennial herbaceous creeper of the Apiaceae family, which grows in tropical regions of Asia, Oceania, Africa and America. It contains several triterpenes, namely, asiatic acid, madecassic acids, asiaticoside and madecassoside. The medical applications of *C. asiatica* involves its anti-inflammatory [9], memory improvement [10], anticancer [11], antihepatoma [12] and antioxidation [13] effects. *C. a*siatica is also recommended for the treatment of dermatoses and skin lesions such as excoriations, hypertrophic scars, eczema, healing wounds, psoriasis and scleroderma[14]. Madecassoside is the main active ingredient of *C. asiatica* with a higher content than other triterpenoid constituents, and its antioxidant capacity has been confirmed [15]. Madecassoside is extracted from *C. asiatica* with a purity of 99%. Some traditional Chinese Medicine doctors have also found its therapeutic effects for external use in the repigmentation of vitiligo and postinflammatory hypopigmentation, but its mechanism of action has not been elucidated. In this study, we evaluated the effects of madecassoside on oxidative stress in human melanocytes, particularly focusing on mitochondria and dendritic morphology, and we discuss its possible mechanism of action.

## RESULTS

### Effect of H_2_O_2_ on normal human melanocyte dendrites

**Figure 1:**
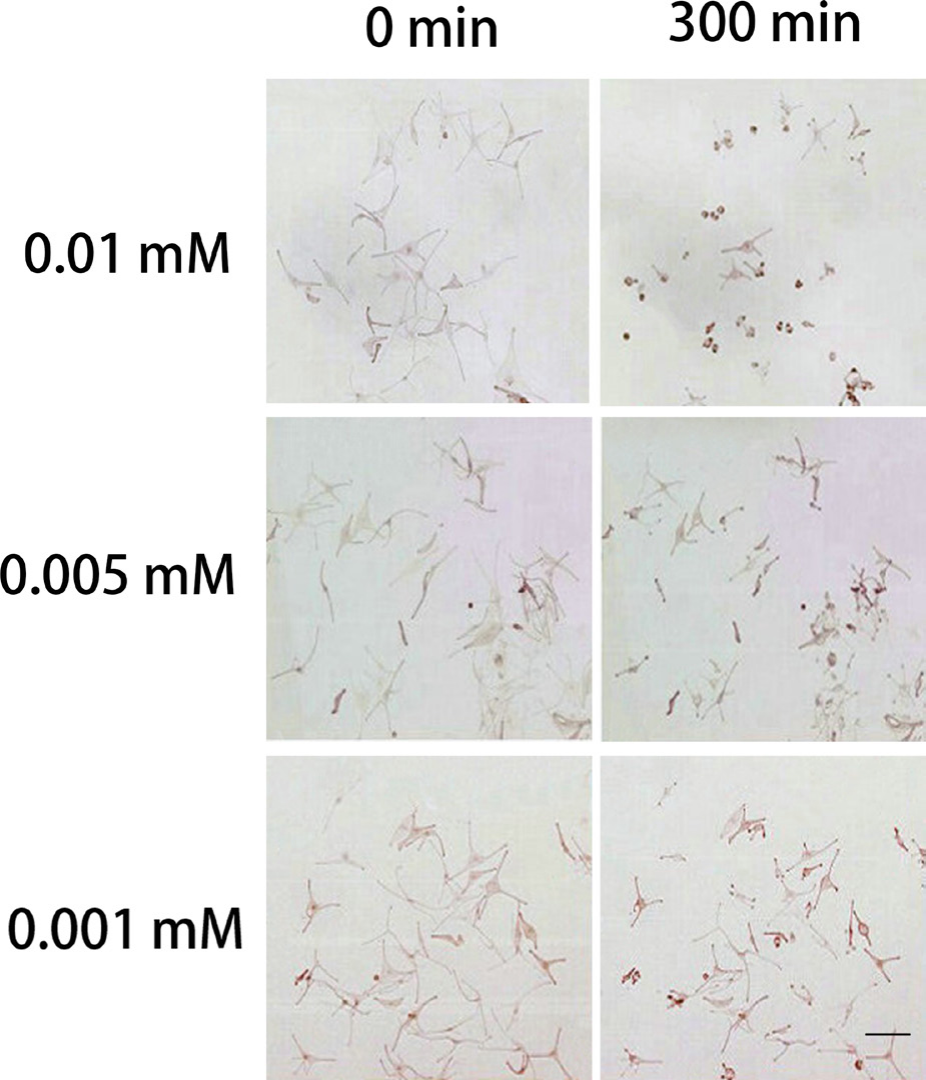
Effects of H_2_O_2_ on normal human melanocytes Images were taken before treatment and at 300 min after treatment with 0.01, 0.005 or 0.001 mM H_2_O_2_ in the culture medium. H_2_O_2_ induced the retraction of dendrites and the detachment of melanocytes when removing the culture medium and rinsing cells to replace the medium. 0.01 mM H_2_O_2_ caused a more obvious dendritic retraction than the other two concentrations of H_2_O_2_. Bar = 200 μm (Original magnification 100×)

The addition of 0.01, 0.005 or 0.001 mM H_2_O_2_ to the culture medium induced the retraction of dendrites and the detachment of melanocytes when replacing the culture medium (Figure 1). Moreover, the dendrites of 0.01 mM H_2_O_2_-treated melanocytes retracted faster than the other two groups (Table 1), and dendrite retraction was observed as early as 5 min after H_2_O_2_ incubation. Considering that a low concentration of H_2_O_2_ would make it rather difficult to observe the obvious dendritic retractions, and would increase factors beyond the control of the experiment, we used 0.01 mM H_2_O_2_ throughout the remainder of these experiments.

**Table 1:** Retraction velocity of melanocytes following H_2_O_2_ treatment (*n* = 60, mean ± SD)

Concentration of H_2_O_2_(mM)	Retraction velocity of melanocytes (μm/min)	*P* value
0.01	0.310 ± 0.021	
0.005	0.199 ± 0.034	0.0007*
0.001	0.189 ± 0.017	0.0003*

*indicates a statistically significant difference (*P <* 0.05) compared with 0.01 mM H_2_O_2_-treated melanocytes. 0.005 mM vs 0.001 mM, *P* = 0.621.

### Madecassoside reduces the retraction of melanocyte dendrites under oxidative stress

**Figure 2:**
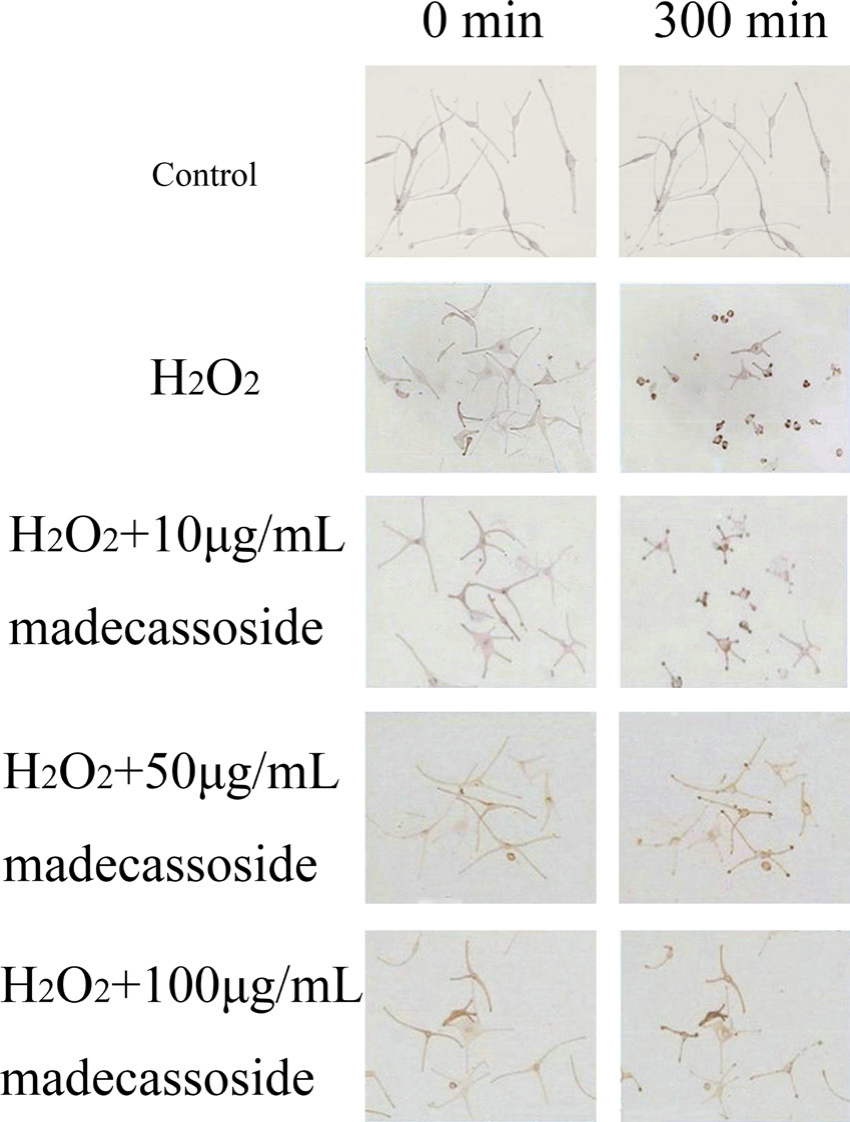
Effect of madecassoside on H_2_O_2_-treated human melanocytes Melanocyte dendrites retracted dramatically after 5 hr when treated only by H_2_O_2_. The addition of madecassoside reduced the dendritic retraction velocities under oxidative stress. The higher the concentration of madecassoside, the greater the suppressive effect on dendritic retractions. Bar = 100 μm (Original magnification 200×).

Changes of dendrites retraction were observed over time after the addition of H_2_O_2_ with or without madecassoside. Dendrites of only H_2_O_2_-treated melanocytes retracted dramatically by 300 min and some dendrites even disappeared. However, the concurrent addition of madecassoside at concentrations of 10, 50 or 100 μg/mL induced only slight dendritic retractions over the same time course (Figure 2), and the retraction velocities of those dendrites were reduced to 0.256 ± 0.021, 0.215 ± 0.017 and 0.167 ± 0.024 μm/min respectively (Table 2). Compared with the retraction velocities in only H_2_O_2_-treated melanocytes (0.310 ± 0.021 μm/min), madecassoside inhibited the retraction of melanocyte dendrites in a concentration-dependent manner.

**Table 2:** Retraction velocity of melanocytes following different treatments (*n* = 100, mean ± SD)

Treatment	Retraction velocity of melanocytes (μm/min)	*P* value
Control	0.019 ± 0.008	0.001*
H_2_O_2_	0.310 ± 0.021	
H_2_O_2_+10 μg/mL madecassoside	0.256 ± 0.021	0.004*
H_2_O_2_+50 μg/mL madecassoside	0.215 ± 0.017	0.003*
H_2_O_2_+100 μg/mL madecassoside	0.167 ± 0.024	0.003*

*indicates a statistically significant difference (*P < 0.05*) compared with H_2_O_2_-treated melanocytes.

### Effect of madecassoside on MMP in melanocytes under oxidative stress

**Figure 3:**
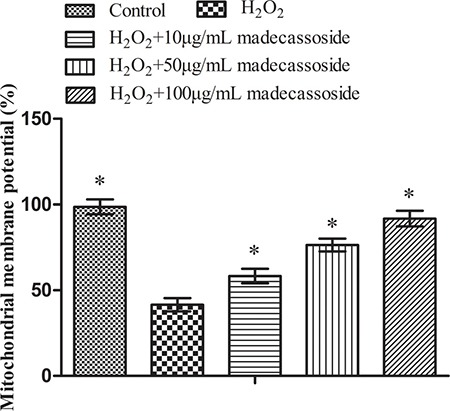
Madecassoside improves the MMP under oxidative stress *P < 0.05 vs. H_2_O_2_ treatment group.

As shown in Figure 3, H_2_O_2_ treatment reduced MMP which decreased from 98.68 ± 3.28% to 41.57 ± 3.91%, but the concurrent treatment with 10, 50 or 100 μg/mL madecassoside rescued MMP to 58.28 ± 5.07%, 76.42 ± 4.06% and 91.87 ± 4.52%, respectively. In addition, there was a significant difference between each madecassoside-treated group. These results revealed that madecassoside and improves MMP in melanocytes under oxidative stress.

### Effect of madecassoside on free intracellular calcium ([Ca^2+^]i) in H_2_O_2_-treated melanocytes

**Figure 4:**
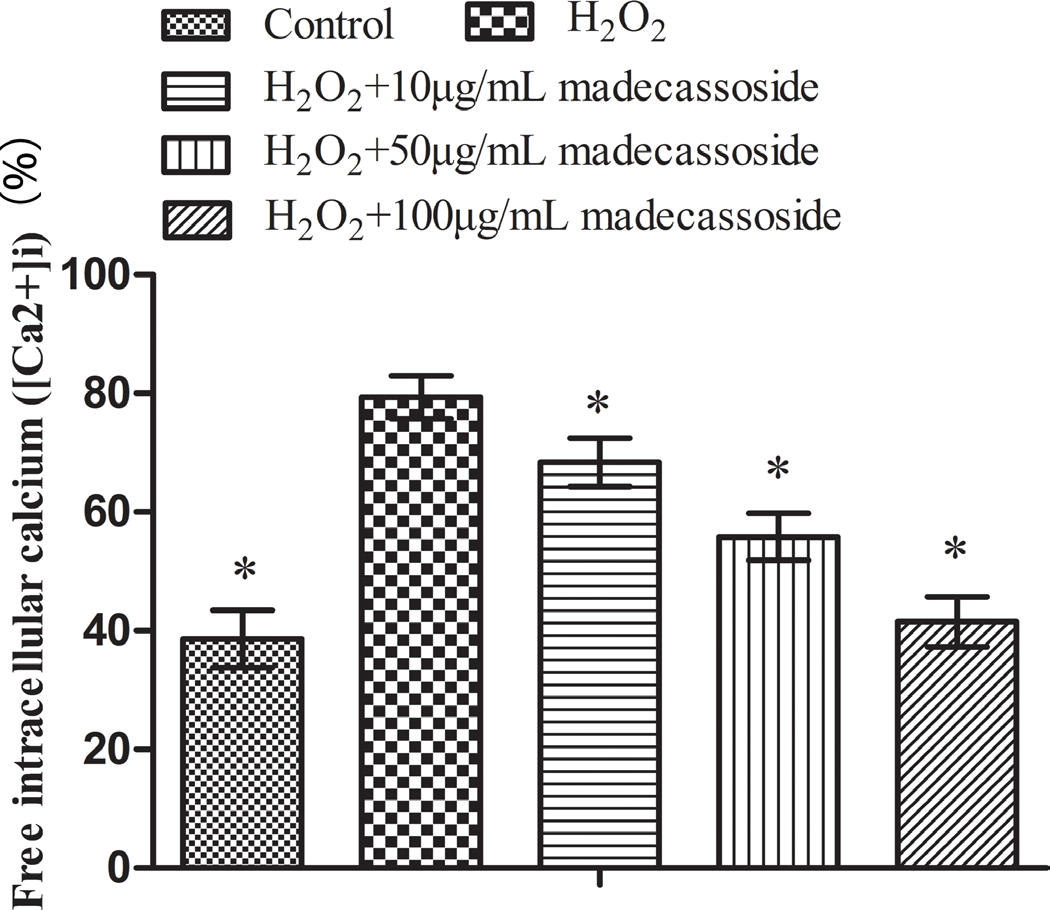
Effect of madecassoside on decreasing the level of free [Ca2+]i in H_2_O_2_-treated melanocytes *P < 0.05 vs. H_2_O_2_ treatment group.

Mitochondria play an important role in maintaining Ca^2+^ homeostasis. To some extent, the disruption of Ca^2+^ homeostasis reflects mitochondrial dysfunction. After exposure to H_2_O_2_, the level of [Ca^2+^]i increased significantly. However, a reduction of [Ca^2+^]i in a concentration-dependent manner was observed in the presence of madecassoside (10, 50 or 100 μg/mL) (Figure 4). These results suggest that madecassoside reduces the accumulation of [Ca^2+^]i and sequentially recovers Ca^2+^ homeostasis in melanocytes under oxidative stress.

### Protective effects of madecassoside on oxidative stress-induced ultrastructural changes in melanocytes

**Figure 5:**
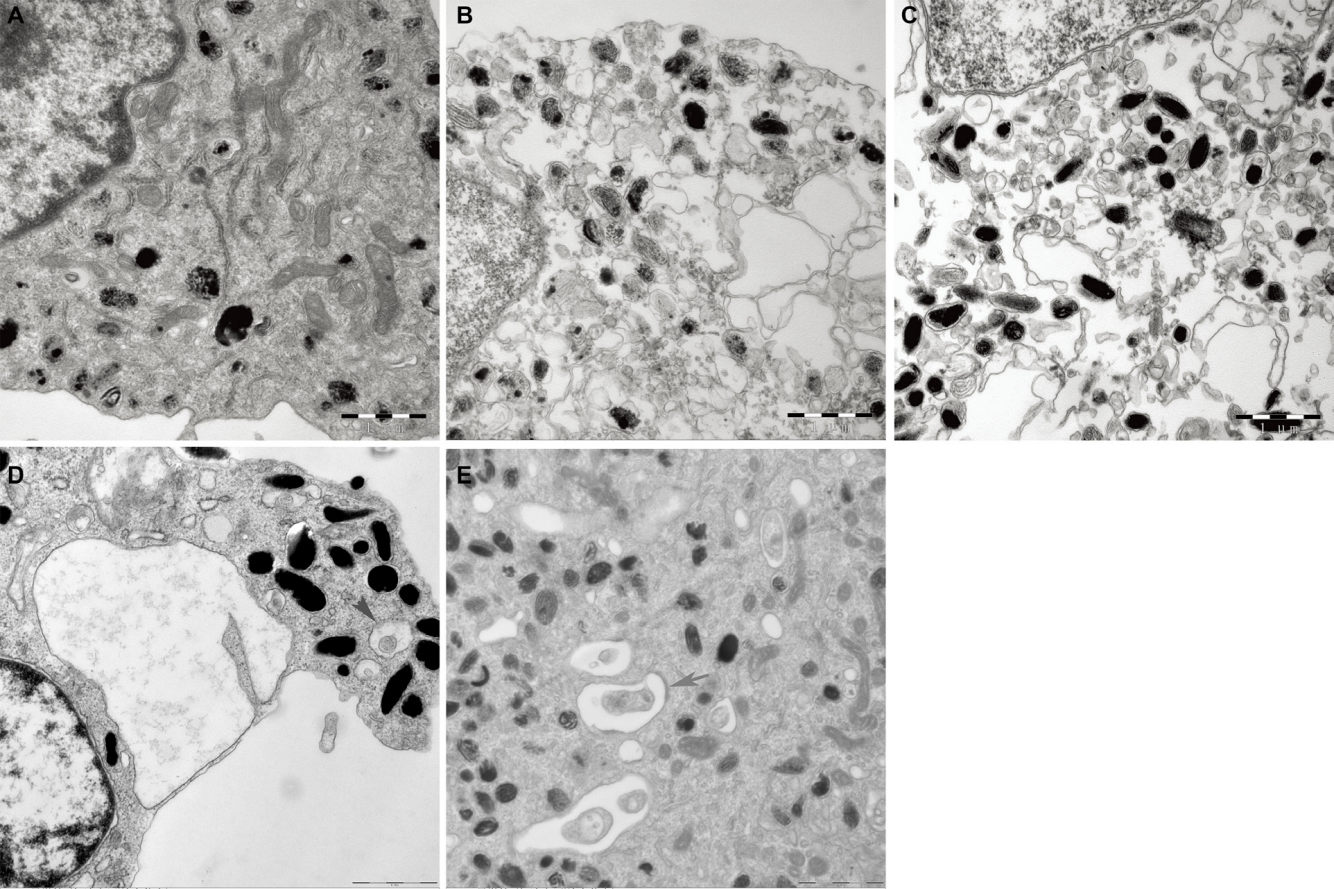
Ultrastructural alterations of mitochondria in melanocytes (**A**) The structure and the number of mitochondria in the untreated control group were quite normal. (**B**) Damaged of mitochondria in H_2_O_2_-treated melanocytes were observed, including disruption of the cytomembrane, as well as swelling and vacuolization of mitochondria. (**C**–**E**) corresponding 10, 50 and 100 μg/mL madecassoside-treated melanocytes, respectively. Madecassoside attenuated the damage of mitochondria caused by H_2_O_2_. Autophagosomes could be also observed (grey arrow).(Original magnification 30,000×).

Some indications of damage in melanocytes were observed by TEM in the H_2_O_2_ treatment group, including the disruption of cytomembrane integrity, a decreased quantity of cristae as well as swelling and vacuolization of mitochondria (Figure 5). However, the degree of mitochondria injury in madecassoside-treated melanocytes was much less than that in H_2_O_2_-treated melanocytes, and a large number of normal mitochondria, even some autophagosomes, could be found.

The stereology results are shown in Table 3. In comparison with the control group, the Vv and Sv of mitochondria in melanocytes treated with H_2_O_2_ were significantly higher, whereas the level of Nv was lower, which indicated the swelling of mitochondria. After exposure to madecassoside, the Vv and Sv decreased, accompanied by an increase of Nv. Remarkable differences were also noted between the H_2_O_2_ and the madecassoside-treated melanocytes for Vv, Sv and Nv with increasing concentrations of madecassoside.

**Table 3:** Stereology analysis of mitochondria after different treatments (*n* = 50, mean ± SD)

Treatment	Nv (μm^-2^)	Sv (‰)	Vv (%)	*P* value	*P* value	*P* value
Control	8.25 ± 0.98	3.66 ± 0.76	5.34 ± 0.95	0.001*	0.002^Δ^	0.002**
H_2_O_2_	3.84 ± 1.03	6.93 ± 0.84	9.13 ± 1.06			
H_2_O_2_+10μg/mL madecassoside	5.73 ± 0.94	5.81 ± 1.01	7.99 ± 0.88	0.004*	0.006^Δ^	0.005**
H_2_O_2_+50μg/mL madecassoside	6.49 ± 0.87	4.68 ± 0.96	6.85 ± 0.78	0.003*	0.004^Δ^	0.003**
H_2_O_2_+100μg/mL madecassoside	7.22 ± 0.95	4.03 ± 0.75	6.27 ± 0.92	0.001*	0.004^Δ^	0.002**

*indicates a statistically significant difference in Nv (*P < 0.05*) compared with H_2_O_2_-treated melanocytes.

^Δ^ indicates a statistically significant difference in Sv (*P < 0.05*) compared with H_2_O_2_-treated melanocytes.

**indicates a statistically significant difference in Vv (*P < 0.05*) compared with H_2_O_2_-treated melanocytes.

### Effect of madecassoside on autophagy activation in H_2_O_2_-treated melanocytes

**Figure 6:**
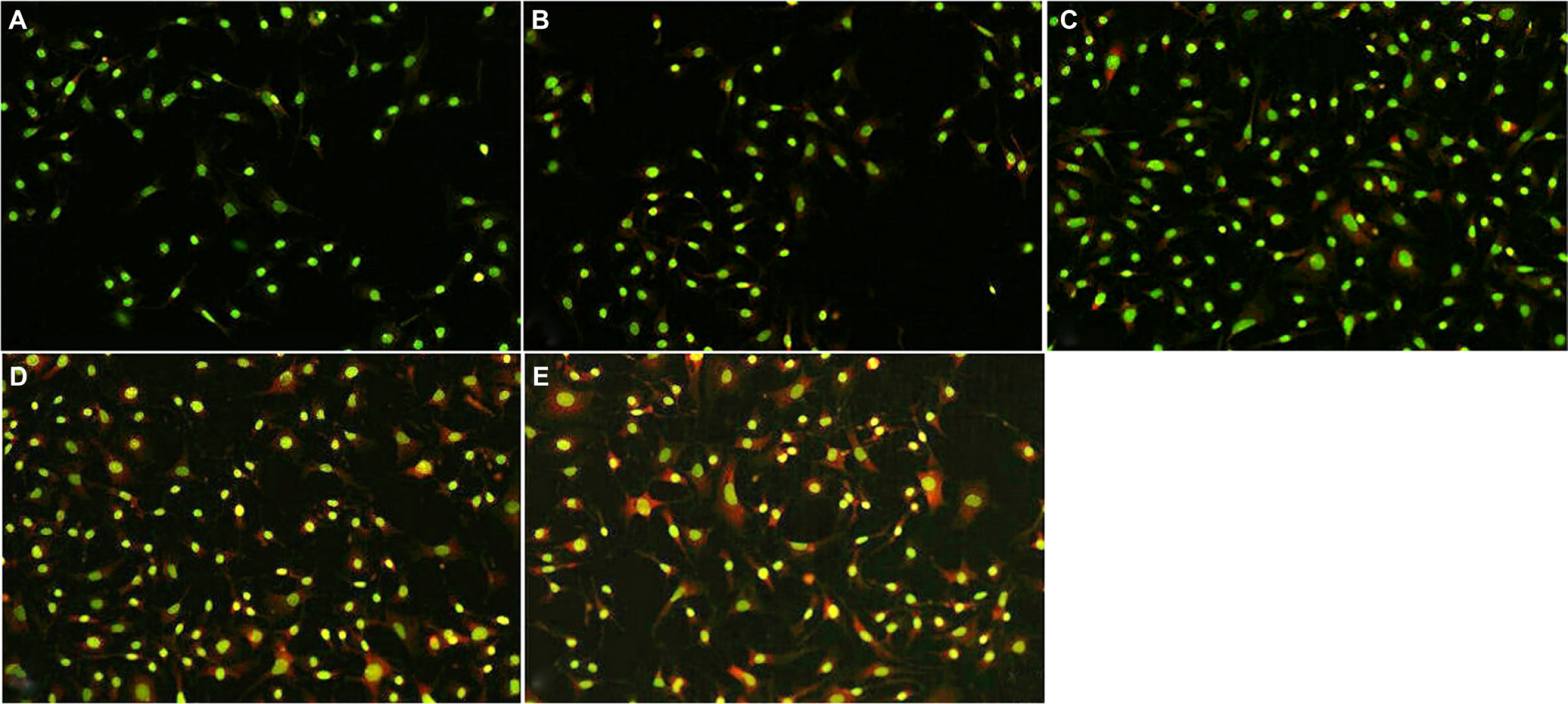
Madecassoside induced autophagy activation in H_2_O_2_-treated melanocytes The number of red fluorescent spots in the cytoplasm of madecassoside-treated melanocytes was increased in a concentration-dependent manner. (**A**) Untreated control group, (**B**) H_2_O_2_-treated melanocytes, and (**C**–**E**) 10, 50 or 100 μg/mL madecassoside-treated melanocytes, respectively. (Original magnification 100×).

AO is a fluorescent substance that is often used to detect the occurrence of autophagy. Madecassoside induced a far stronger red punctate fluorescence in the cytoplasm of H_2_O_2_-treated melanocytes in comparison with the only H_2_O_2_-treated melanocytes (Figure 6). Moreover, the number of AO-positive acidic vesicular organelles (AVOs) in the madecassoside-treated cells was increased in a concentration-dependent manner.

### Madecassoside increases the LC3-II/LC3-I ratio in melanocytes under oxidative stress

**Figure 7:**
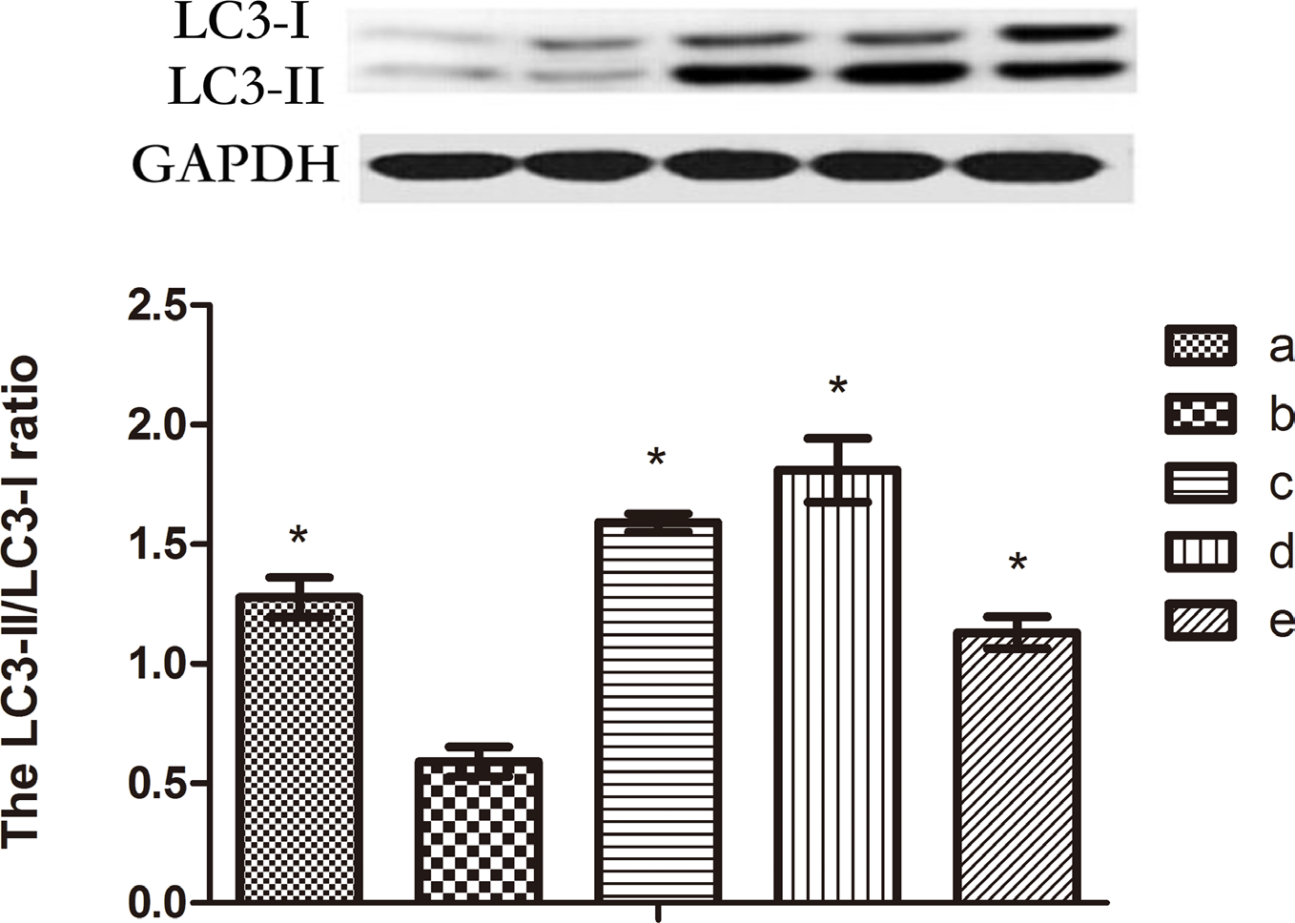
Western blot analysis of LC3 The protein level of the LC3-II/LC3-I ratio increased significantly following madecassoside treatment, where compared with the H_2_O_2_ treatment group. (**A**) Untreated control group, (**B**) H_2_O_2_-treated melanocytes, and (**C**–**E**) 10, 50 or 100 μg/mL madecassoside-treated melanocytes, respectively. *P < 0.05 vs. H_2_O_2_ treatment group.

LC3-II is a significant marker of autophagosomes in mammalian cells[17]. Levels of LC3-II and LC3-I in H_2_O_2_-treated melanocytes were up-regulated significantly by simultaneous incubation with 10, 50 or 100 μg/mL madecassoside. The relative ratio of LC3-II/LC3-I also increased markedly in a concentration-dependent manner compared with H_2_O_2_-treated melanocytes (*P <* 0.05, Figure 7). Hence, madecassoside activates the LC3-I to LC3-II conversion in melanocytes under oxidative stress.

## DISCUSSION

Vitiligo is a multifactorial polygenic disorder with a complex pathogenesis. Recently, melanocytorrhagy arising from defective cell-cell adhesion has been proposed as a pathomechanism for vitiligo, which considers nonsegmental vitiligo as a disease caused by the chronic detachment and transepidermal loss of melanocytes[18]. Kumar et al. demonstrrated that the dendrites of melanocytes from patients with unstable nonsegmental vitiligo are small with clubbed ends, and show a significantly lower adhesion to collagen type IV compared with control and stable vitiligo melanocytes [19]. These observations support the melanocytorrhagic theory. Dendrites are important not only for melanosome transfer, but also dramatically increase the adhesion and anchoring of melanocytes within the basal layer of the epidermis. Routine observation of melanocytes in culture indicates that dendrite retraction comes first, before cell detachment and eventual death. Increasing evidence shows that oxidative stress caused by H_2_O_2_ is a key factor in the onset and progression of vitiligo [1]. More importantly, it has been suggested that the loss of melanocytes in vitiligo may be the result of oxidative stress and melanocytorrhagy acting in unison. ROS could induce the loss of dendricity of melanocytes, which might not only affect melanosome transfer but could also exaggerate traumatic transepidermal loss of genetically adhesion-deficient melanocytes [5]. Additionally, extracellular Ca^2+^ is essential for dendrite outgrowth and attachment. It is well known that mitochondria play an essential role in maintaining calcium homeostasis, and they are the main source of ROS, as well as an unifying target of ROS, so the status of mitochondrial structure and function may be strongly related with the onset of vitiligo.

In this study, 0.01 mM H_2_O_2_ was employed to induce of oxidative stress in human melanocytes, and we found an obvious retraction of dendrites, structural damage and functional disruption of mitochondria, including a decreased quantity of their cristae, swelling and vacuolization a decrease of MMP and the accumulation of [Ca^2+^]i in melanocytes under oxidative stress. It has been reported that the major mechanism for mitochondrial Ca^2+^ transport is primarily via the mitochondrial Ca^2+^ uniporter (MCU) and Ca^2+^ uptake is driven by the MMP [20]. Accordingly, we speculate that the stimulation of ROS might lead to the opening the mitochondrial permeability transition pore (MPTP) and MMP depolarization, followed by the disruption of Ca^2+^ homeostasis, suggesting that dendrite retraction might be related to the overload of [Ca^2+^]i.

Our results show that the detrimental effects of H_2_O_2_ on melanocytes are alleviated by madecassoside in a concentration-dependent manner. Our observations showed that madecassoside remarkably reverses the decrease of MMP and inhibits the accumulation of [Ca^2+^]i induced by oxidative stress. In addition, madecassoside protects the structure of mitochondria against oxidative stress. Mitochondrial swelling and/or vacuolization was only rarely observed and a large number of normal mitochondria could be found by TEM. The stereology data revealed that Vv and Sv of mitochondria in melanocytes treated with H_2_O_2_ were much higher than those in the untreated control melanocytes and decreased following treatment with increasing concentrations of madecassoside, and the changes of Nv were contrary to the Vv and Sv. Taken together, the underlying mechanism involved in the protective effect of madecassoside on melanocyte dendrites might be its antioxidative impact including the protection of MMP and the recovery of Ca^2+^ homeostasis.

Autophagy is a cellular degradation pathway essential for survival. As a protective mechanism, autophagy safeguards organisms against normal and pathological aging by regulating the turnover of dysfunctional organelles and proteins [21]. It is thought that damaged or excess mitochondria are eliminated by autophagy, and TEM revealed the formation of autophagic vacuoles in melanocytes treated with madecassoside. Accordingly, we speculate that madecassoside might play a role in the antioxidant defense against melanocyte loss through autophagy. To further investigate that possibility, we also examined the effect of madecassoside treatment on the formation of AVOs in melanocytes using fluorescence microscopy. As shown in Figure 6, madecassoside-treated melanocytes displayed primarily red fluorescence in their cytoplasm with minimal green fluorescence, indicating the abundance of AVOs. Futher, the LC3-II/LC3-I ratio was analyzed using western blots. During autophagy, the cytosolic form of LC3 (LC3-I) is cleaved and lipidated to form the phosphatidylethanolamine-conjugated form (LC3-II) that is recruited to the autophagosomal membrane [22]. The degree of autophagosome formation can be measured using the LC3-II/LC3-I ratio and/or by the expression level of LC3-II. We observed that madecassoside-treated melanocytes had a higher LC3-II expression and also an increased LC3-I conversion into LC3-II compared to H_2_O_2_-treated melanocytes, suggesting an increased activation of autophagy. This proves that autophagy occurs in melanocytes following treatment with madecassoside against oxidative stress.

## MATERIALS AND METHODS

### Cell culture

Human melanocytes were obtained from normal foreskins of 10 young healthy male subjects aged 7–10 years old (skin phototype III or IV, Fitzpatrick classification) after circumcision, according to the method of Swope et al. [16]. This study was conducted after signed consent of each participant in accordance with the guidelines in the Declaration of Helsinki Principles and was approved by the local Ethics Committees of the First Affiliated Hospital of Nanjing Medical University. Briefly, each foreskins was sterilized in 70% ethanol, cut into small pieces (4 × 4 mm), and then incubated in 5 mg/mL Dispase II (Sigma, St Louis, MO, USA) overnight at 4°C. The epidermis was separated using forceps and was further digested with 0.25% trypsin/EDTA (Gibco, Carlsbad, CA, USA) at 37°C for 30 min. After centrifugation, cells were resuspended in growth medium Medium 254 with human melanocyte growth supplement (Cascade Biologics, Portland, OR, USA) and were cultured in a humidified incubator with 5% CO_2_/95% air at 37°C. During the primary culture of human melanocytes, 200 mg/mL G418 (geneticin sulfate, Duchefa, Haarlem, Netherlands) was added to the growth medium to suppress the proliferation of fibroblasts. The culture medium was changed every other day thereafter until the cells became confluent. All experiments were performed using human melanocytes at passages 2–5. Melanocytes were identified by staining with the antibody HMB45 and Masson-Fontana (both from Life Technologies, Carlsbad, CA, USA) as markers of PMEL17 and melanin, respectively.

### Observation of melanocytes dendrites

To determinate the optimal concentration of H_2_O_2_ to observe melanocytes dendrites under oxidative stress conditions *in vitro*, the cells were photographed using a light microscope (Olympus BX51, Aizu, Japan) before treatment and every 5 min after treatment with various concentrations of H_2_O_2_ (0.01, 0.005, 0.001 mM, Nanjing Chemical Reagent Factory, Nanjing, China). The lengths of dendrites were measured using AutoCAD Software (Autodesk, Inc. San Rafael, CA, USA). Twenty melanocytes in each group were randomly selected to calculate the retraction velocity according to Eq. (1).

$$\text{Retraction velocity}=\left({\text{Length}}_{\text{initial}}-{\text{Length}}_{\text{final}}\right)/\text{time}\left(\mu \text{m}/\text{min}\right)$$ 1

Subsequently, cells were treated with H_2_O_2_ (0.01 mM) and final concentrations of madecassoside (10, 50, 100 μg/mL) concurrently, and the assessment of retraction velocity of melanocyte dendrites was performed as noted above. Cells cultured without madecassoside or H_2_O_2_ served as a control group. Madecassoside powder was a gift from Shanghai Shyndec Pharmaceutical Co. Ltd. (Shanghai, China)

### Determination of mitochondrial membrane potential (MMP)

JC-1 dye exhibits potential dependent accumulation in mitochondria, indicated by a change in fluorescence emission from green to red. Consequently, mitochondrial depolarization is indicated by a decrease in the red/green fluorescence intensity ratio. In brief, human melanocytes were grown in 6-well plates to approximately 90% confluence, and then were treated with H_2_O_2_ (0.01 mM) and/or a variety of concentrations of madecassoside (10, 50, 100 μg/mL) for 5 hr. A total of 0.5 mL of each cell suspension collected from the different groups was added to 0.5 mL JC-1 solution (Sigma, St Louis, MO, USA) followed by incubation at 37°C for 15 min. Each cell suspension was then centrifuged at 60 g for 5 min to remove the supernatant. The cell pellets were resuspended in JC-1 solution and analyzed by flow cytometry using a FACScan and Cell Quest software (Becton Dickinson, Mountain View, CA, USA). Mean fluorescence intensity values for FL1 (green fluorescence) and FL2 (red fluorescence) were obtained, and the ratio of Red/Green Fluorescence intensity values provided an estimate of the MMP (ΔΨm).

### Intracellular calcium measurements by flow cytometry

Levels of free intracellular calcium ([Ca^2+^]i) were measured using the cell-permeable calcium-sensitive fluorescent dye Fluo-3/AM (Sigma, St Louis, MO, USA). After treatment with H_2_O_2_ (0.01 mM) and/or final concentrations of madecassoside (0, 10, 50, 100 μg/mL) for 5 hr, cells were collected and incubated with 5 μM Fluo-3/AM for 35 min at 37°C. The cells were washed twice with PBS, and the fluorescence intensity of Fluo-3/AM probes was analyzed by flow cytometry using Cell Quest software (Becton Dickinson, Mountain View, CA) with excitation at 488 nm and emission at 530 nm.

### Transmission electron microscopy (TEM)

Human melanocytes were treated with H_2_O_2_ (0.01 mM) and/or a variety of final concentrations of madecassoside (10, 50, 100 μg/mL) for 5 hr and were harvested. Cell pellets were washed twice with PBS and fixed with 3% glutaraldehyde at 4 °C overnight. Thereafter they were postfixed with PBS containing 1% (v/v) OsO_4_, dehydrated in a series of graded ethanol (70–100%), embedded in Durcopan (Durcopan, Fluka Chemie, Buchs, Switzerland) and sectioned using a LEICA Ultracut UCT ultramicrotome (Leica Microsystems, Wetzlar, Germany). Ultrathin sections were stained with uranyl acetate and lead citrate, and were examined using TEM (JEOL Ltd., Tokyo, Japan). Images were captured and input into the electron microscope image analysis system for morphological quantitative analysis of melanocytes and mitochondria. The mean long axis, minor axis, circumference and area of 10 melanocytes in each specimen were measured. The numeral density (Nv), surface density (Sv) and volume density (Vv) of mitochondria were calculated by Eq. (2), Eq. (3) and Eq. (4), respectively.

$$N_{v}=\pi N_{x}/(A_{c}\cdot 4\sqrt{ab}$$ 2

$$S_{v}=4B_{x}/\left(\pi \cdot A_{c}\right)$$ 3

$$V_{v}=V/V_{c}$$ 4

N_x_: number of mitochondria in the cytoplasm, *A*_c_: area of the cytoplasm, *a*: long axis of mitochondria, *b*: minor axis of mitochondria, *B*_x_: circumference of mitochondria, *V*: mitochondrial volume, *V*_c_: cytoplasmic volume

### Acridine orange (AO) staining

Melanocytes for AO fluorescent staining were cultured in 6-well plates at a density of 1 × 10^5^ cells/well. After treatment with various concentrations of madecassoside (0, 10, 50, 100 μg/mL) as well as 0.01 mM H_2_O_2_ for 5 hr, cells were incubated in PBS with 1 μg/mL AO (Sigma, St. Louis, MO, USA) for 15 min at room temperature in the dark, and then were washed twice by PBS. Acidic vacuoles were detected using a fluorescence microscope (Nikon TE2000-U, Shizvoka, Japan). The protonated form of AO accumulates in acidic compartments and forms aggregates, which are characterized by red fluorescence.

### Western blot analysis

Melanocytes were inoculated in 6-well plates at 2 × 10^5^ cells/well, and were concurrently treated with H_2_O_2_ (0.01 mM) and madecassoside at final concentrations of 0, 10, 50, 100 μg/mL for 5 hr. Each well was then harvested by rinsing twice with cold PBS, and was lysed in RIPA buffer containing protease inhibitors. Lysates were cleared by centrifugation at 290g for 15 min and protein concentrations were determined using a Bradford protein assay kit (Bio-Rad Laboratories, Hercules, CA, USA). Equal amounts of protein were resolved by SDS-PAGE and were transferred to nitrocellulose membranes (Schleicher & Schuell, Keene, NH, USA) by electroblotting. Membranes were then blocked and incubated at 4°C overnight with primary antibodies [rabbit anti-LC3 (1:1000, L7543, Sigma, St. Louis, MO, USA) and mouse anti-GAPDH (1:1000, G8795, Sigma, St. Louis, MO, USA)]. Subsequently, the membranes were washed and incubated for 1–2 hr at room temperature with horseradish peroxidase-conjugated anti-rabbit or anti-mouse as the secondary antibody (1:1000, Jackson ImmunoResearch Lab., West Grove, PA, USA). Specific signals were detected with the ECL detection reagent (Pierce, Rockford, IL USA). The quantification of LC3 was analyzed by Quantity One software (Bio-Rad, Hercules, CA, USA).

### Statistics

Data are expressed as means ± SD. All statistical analyses were performed with SPSS 16.0 software (International Business Machines). Comparisons among 3 or more groups were assessed via one-way analysis of variance, and differences among means were analyzed using Least significant difference (LSD). A *P <* 0.05 is considered significant.

### Ethical approval

All procedures performed in studies involving human participants were in accordance with the ethical standards of the institutional and/or national research committee and with the 1964 Helsinki declaration and its later amendments or comparable ethical standards. This article does not contain any studies with animals performed by any of the authors.
